# Ambulance dispatch calls attributable to influenza A and other common respiratory viruses in the Netherlands (2014‐2016)

**DOI:** 10.1111/irv.12731

**Published:** 2020-05-14

**Authors:** Susana Monge, Janneke Duijster, Geert Jan Kommer, Jan van de Kassteele, Thomas Krafft, Paul Engelen, Jens P. Valk, Jan de Waard, Jan de Nooij, Annelies Riezebos‐Brilman, Wim van der Hoek, Liselotte van Asten

**Affiliations:** ^1^ Centre for Infectious Disease Control Netherlands (CIb) National Institute for Public Health and the Environment (RIVM) Bilthoven The Netherlands; ^2^ European Programme for Intervention Epidemiology Training (EPIET) European Centre for Disease Prevention and Control, (ECDC) Stockholm Sweden; ^3^ Centre for Nutrition, Prevention and Health Services (VPZ) National Institute for Public Health and the Environment (RIVM) Bilthoven The Netherlands; ^4^ Faculty of Health, Medicine and Life Sciences Maastricht University Maastricht Centre for Global Health Maastricht The Netherlands; ^5^ Meldkamersupport Utrecht The Netherlands; ^6^ Dispatch Center Regional Ambulance Services Noord Nederland Leiden The Netherlands; ^7^ Department of Anesthesiology University of Groningen University Medical Center Groningen Groningen The Netherlands; ^8^ Regional Ambulance Service Hollands Midden Leiden The Netherlands; ^9^ Department of Microbiology University Medical Centre Utrecht Utrecht University Utrecht The Netherlands

**Keywords:** adenovirus, ambulance, coronavirus, influenza, respiratory syncytial virus, rhinovirus

## Abstract

**Background:**

Ambulance dispatches could be useful for syndromic surveillance of severe respiratory infections. We evaluated whether ambulance dispatch calls of highest urgency reflect the circulation of influenza A virus, influenza B virus, respiratory syncytial virus (RSV), rhinovirus, adenovirus, coronavirus, parainfluenzavirus and human metapneumovirus (hMPV).

**Methods:**

We analysed calls from four ambulance call centres serving 25% of the population in the Netherlands (2014‐2016). The chief symptom and urgency level is recorded during triage; we restricted our analysis to calls with the highest urgency and identified those compatible with a respiratory syndrome. We modelled the relation between respiratory syndrome calls (RSC) and respiratory virus trends using binomial regression with identity link function.

**Results:**

We included 211 739 calls, of which 15 385 (7.3%) were RSC. Proportion of RSC showed periodicity with winter peaks and smaller interseasonal increases. Overall, 15% of RSC were attributable to respiratory viruses (20% in out‐of‐office hour calls). There was large variation by age group: in <15 years, only RSV was associated and explained 11% of RSC; in 15‐64 years, only influenza A (explained 3% of RSC); and in ≥65 years adenovirus explained 9% of RSC, distributed throughout the year, and hMPV (4%) and influenza A (1%) mainly during the winter peaks. Additionally, rhinovirus was associated with total RSC.

**Conclusion:**

High urgency ambulance dispatches reflect the burden of different respiratory viruses and might be useful to monitor the respiratory season overall. Influenza plays a smaller role than other viruses: RSV is important in children while adenovirus and hMPV are the biggest contributors to emergency calls in the elderly.

## 1 INTRODUCTION

Surveillance of respiratory viruses is mainly centred on influenza, for which robust systems have been developed in most countries, generally based on sentinel networks of General Practitioners (GPs, primary care).^1^ Comparable surveillance systems do not exist for other respiratory viruses, despite the increasing interest and leadership of the World Health Organization (WHO) in expanding surveillance for respiratory syncytial virus (RSV) now that a vaccine may become available.^2^ For most viruses, surveillance is limited to laboratory‐based counts, often with unknown denominator, low representativeness or lack of standard sampling criteria.

WHO encourages surveillance of severe acute respiratory infections (SARI) in the context of the Pandemic Influenza Severity Assessment program.^1^ This is fundamental to determine the severity of circulating viruses, their pressure on healthcare services and the groups most at risk of severe outcomes. Surveillance of severe infections requiring secondary care is much less developed than surveillance in primary care. In Europe, a few countries have established hospital‐based surveillance based on syndromic SARI, laboratory confirmed cases or a combination of both.^3^, ^4^ In the Netherlands, a pilot involving three hospitals has been running since 2015.^3^ Syndromic surveillance using ready‐to‐use data has also been explored, mainly in emergency rooms.^5^, ^6^, ^7^ Few initiatives have used ambulance data^5^, ^8^, ^9^, ^10^ or ambulance dispatch centre data.^5^, ^8^, ^9^, ^11^

Ambulance dispatch centres could be an alternative source of readily available data to monitor the occurrence of severe respiratory infections. During the triage process, information is collected and recorded in real time, including the chief symptom in very broad categories, as their objective is to rapidly assign an urgency level and prioritize resources. A recent study in the Netherlands has shown how the variability in respiratory syndromes is correlated with ILI from sentinel GP surveillance,^12^ making it a potential source for syndromic surveillance. However, not all respiratory viruses will result in ILI, and although the ILI case definition focuses on detecting influenza infections, ILI can be caused by a wide range of viruses.

In this study, we aimed to assess to what extent ambulance dispatches reflect the activity of different respiratory viruses in order to advance our understanding of their use for the surveillance of severe acute infections by different respiratory viruses. Specifically, we evaluated the association of syndromes compatible with respiratory infections in ambulance dispatches with trends in detections of influenza A, influenza B, RSV, rhinovirus, adenovirus, coronavirus, parainfluenza and human metapneumovirus (hMPV).

## 2 METHODS

The Netherlands is divided into 25 Regional Ambulance Services (RAV) served by 21 dispatch centres, half of which use the Advanced Medical Priority Dispatch System (AMPDS, Priority Dispatch Cooperation) for triage. The AMPDS is a structured interrogation script that results in a triage code containing the chief symptom and a level of urgency: A1 (immediately life‐threatening, ambulance to reach within 15 minutes) or A2 (urgent but not life‐threatening, reach within 30 minutes). Calls coded as urgency B correspond to planned transports that do not undergo triage.

We included calls from four dispatch centres using AMPDS, and covering 4.2 million people in six RAV: Hollands Midden, Brabant Midden‐West, Brabant Noord, Groningen, Friesland and Drenthe. These include 51% of the population covered by centres using AMPDS, and 25% of the population in the Netherlands. Included centres provided their automatically generated databases from 1 January 2014 up to 31 December 2016, except one centre that implemented AMPDS starting on 24 May 2014 and provided data thereafter. Our data included two complete epidemiologic years (from week 27 to week 26 of the following year: 2014/15 and 2015/16) and two incomplete years: weeks 1‐26, 2014, for epidemiologic year 2013/14 and weeks 27‐52, 2016, for epidemiologic year 2016/17.

We focused our analysis to A1 urgency calls, as we previously found these to have a stronger association with ILI.^12^ These calls may better capture variations in acute severe infections by respiratory viruses and be a valid source for their surveillance.

Calls with triage codes that were potentially compatible with respiratory infections (Table 1) were grouped as respiratory syndrome calls (RSC) and aggregated weekly. Age and sex were also retrieved. A waiver for full medical ethical review was obtained from the Medical Ethical Committee at University Medical Center Utrecht (Ref.WAG/mb/16/01/6181). Data were anonymized, and individuals were not identifiable.

**Table 1:** AMPDS triage codes included in the definition of respiratory syndrome for this study

Code^a^	Description	n	%
6c1	Abnormal breathing	88	0.57
6d2	Abnormal breathing, troubles speaking between two breaths	12 318	80.06
6d3	Abnormal breathing, change in skin colour	204	1.33
6d4	Abnormal breathing, sweaty	1900	12.35
26c2	Sick person, abnormal breathing	875	5.69
Total		15 385	100.00

^a^ The first letter of the code indicates the protocol: 6 is “Breathing problems,” 26 is “Sick person.”

### 2.1 Respiratory virus data

The number of respiratory virus identifications was obtained from the Weekly Sentinel Surveillance System of the Dutch Working Group on Clinical Virology. Twenty‐one virological laboratories voluntarily provide aggregated weekly number of diagnoses; individualized information such as age or sex is not provided. Also, no distinctions are made between primary or secondary care, or different diagnostic methods, although currently the majority use molecular methods or rapid tests.^13^ We included weekly reports of influenza A, influenza B, rhinovirus, RSV, adenovirus, coronavirus, parainfluenza and hMPV.

### 2.2 Statistical analysis

We analysed weekly RSC as a proportion of the total number of calls overall, by age group and by time of the day: office hours (9:00‐16:59, Monday‐Friday) vs out‐of‐office hours. Plotted time series were smoothed using a 5‐week moving average (current ±2 weeks).

We estimated how much of the RSC were potentially attributable to different respiratory viruses. The weekly number of RSC (numerator) relative to the total number of calls of A1 urgency (denominator) was modelled using a binomial generalized linear model with identity link function. Being an additive model, the resulting coefficients are interpreted as differences in proportions, that is the increase in the proportion points of calls that are RSC per each unit increase in the independent variables. The coefficients were further multiplied by 100 to represent the increase in percentages.

The presence of a linear time‐trend and periodic patterns was evaluated using week number and sine and cosine terms with periodicities of 1 year, a half year, third or fourth of a year. They were added in a stepwise forward manner if statistically significant. Pairs of sine and cosine always entered or exited simultaneously. The combination of significant linear and periodic terms plus the intercept was considered as a baseline (RSC not attributed to respiratory viruses).

Respiratory viruses were sequentially added to the model baseline; effects were calculated per increase in 100 virus detections. Because the trends in RSC might coincide, precede or lag behind the trends in viruses reports, we considered virus reports either in the current week, or lagged up to 4 weeks to the right, that is future in time (+lags), or 4 weeks to the left, that is backwards in time (−lags), for a total of 9 time‐lagged variables of each virus. When building the models, the time lag with the lowest *P*‐value was selected, and only one time lag per virus was allowed.

Because one of the viruses with the highest interest in monitoring its severity is influenza A (due to shifts, drifts and its pandemic potential), we forced it into the model, unless its coefficient was negative due to biological implausibility. Subsequently, other viruses were added if statistically significant, had a positive coefficient and did not revert to negative the coefficients of viruses previously added to the model. Finally, because the influenza epidemic size and severity varies by season, an interaction between influenza A and an indicator variable for the epidemiologic year (from week 27 to week 26 of the following year) was tested and retained if *P* < .05. The indicator variable itself was not included, as we wanted to attribute differences between years to influenza A.

Model assumptions and absence of remaining seasonality and autocorrelation were assessed by residuals diagnostics. We used R, version 3.4.0.

## 3 RESULTS

**Figure 1:**
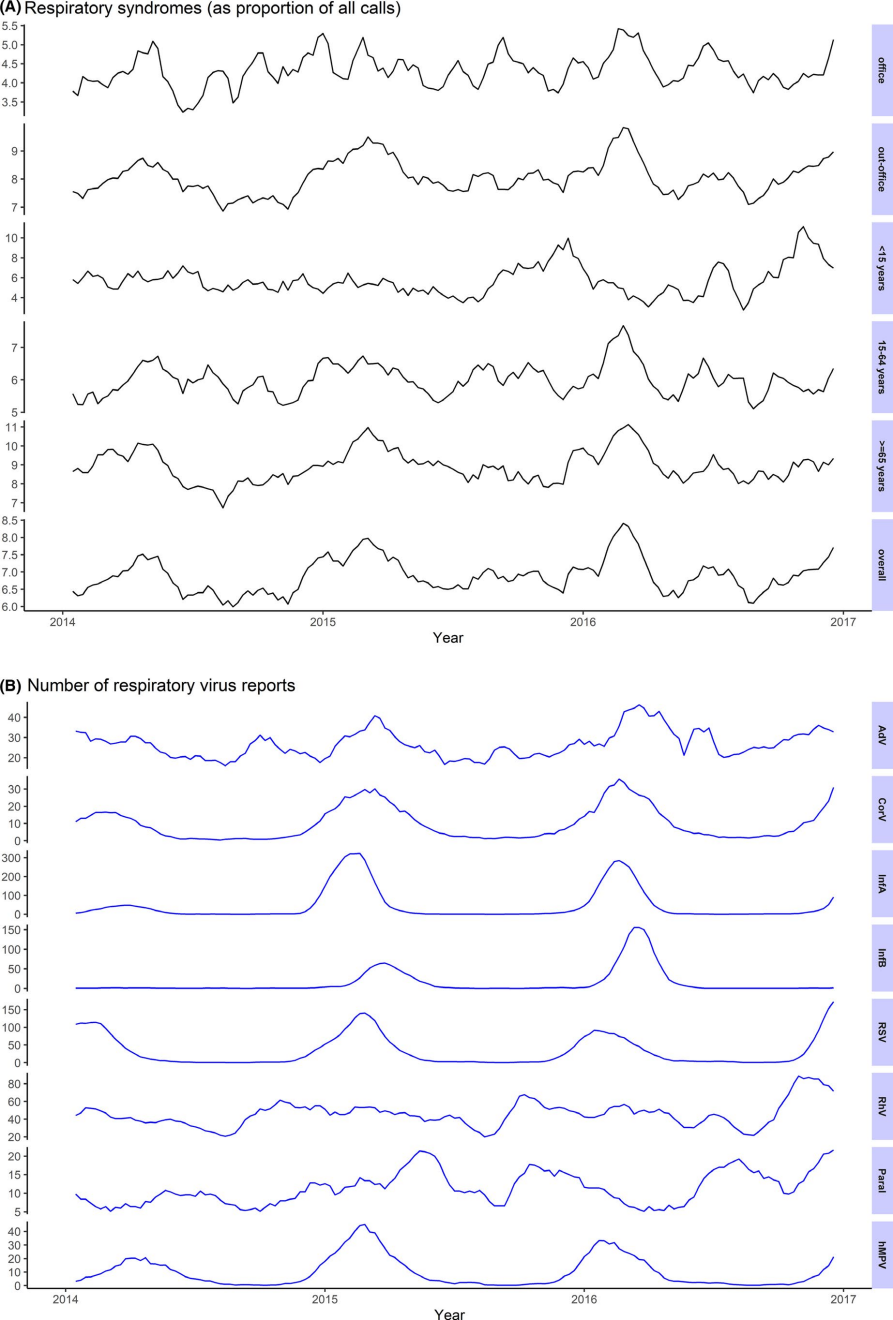
Weekly number of respiratory syndromes and positive laboratory test for respiratory viruses from the weekly sentinel surveillance system of the Dutch working group on clinical virology; 5‐week moving average

Of a total 278 390 dispatch calls between 2014 and 2016, 211 739 (76%) had A1 urgency level and were included; 15 385 (7.3%) were classified as RSC (vs 5.7% in the excluded A2‐urgency calls). The proportion of RSC was slightly lower in the year 2015/16 and higher in people ≥65 years, out‐of‐office hours and in one of the call centres (Table 2). The most frequent triage code among RSC was “Abnormal breathing, troubles speaking between two breaths” (Table 1). Weekly average number of RSC was 98 (range 58‐138), which corresponds to 2.3 calls per 100 000 inhabitants every week. The proportion of RSC showed a periodic pattern peaking in winter, with lower interseasonal peaks (Figure 1). The periodicity was evident in out‐of‐office hours and people ≥65 years, but the pattern was less clear in other groups and, in children <15 years, the peak occurred earlier.

**Table 2:** Number of total ambulance dispatch calls of A1 urgency level and calls with a respiratory syndrome

	Total calls	Calls with respiratory syndrome		
		n	% calls	*P*‐value
Call centre				
Hollands Midden	29 821	2144	7.2	<.001
Brabant Noord	32 976	2345	7.1	
Brabant Midden‐West	60 689	4752	7.8	
Noord Nederland	88 253	6144	7.0	
Age group				
<15 y	11 522	757	6.6	<.001
15‐64 y	101 871	6322	6.2	
≥65 y	69 280	6753	9.8	
Unknown	25 351	1411	5.6	
Sex				
Males	74 078	5306	7.2	.319
Females	62 536	4612	7.4	
Unknown	75 125	5467	7.3	
Epidemiologic year				
2013/14	28 983	2233	7.7	<.001
2014/15	71 049	5298	7.5	
2015/16	75 046	5178	6.9	
2016/17	36 661	2676	7.3	
Time of the day				
Out‐of‐office hours	146 417	12 393	8.5	<.001
Office hours	65 322	2992	4.6	
Total	211 739	15 385	7.3	

Among the included respiratory viruses, the most frequently reported were rhinovirus and influenza A, followed by RSV (Table 3). Most viruses had a periodic pattern similar to RSC, peaking in winter, except for rhinovirus and parainfluenza, which had a less distinct pattern with peaks in the autumn or the spring (Figure 1). Adenovirus reports showed smaller interseasonal peaks in addition to winter peaks.

**Table 3:** Number of positive laboratory tests for respiratory viruses from the Weekly Sentinel Surveillance System of the Dutch Working Group on Clinical Virology

Respiratory viruses	Total	Number by season				Number by week	
	number	wk 1‐26, 2014^a^	2014/15	2015/16	wk 27‐52, 2016^a^	Mean	Range
Rhinovirus	7186	1084	2299	2370	1433	46	(16‐104)
Influenza A	7179	577	3350	2718	534	46	(0‐364)
Respiratory Syncytial virus	5443	1363	1690	1285	1105	35	(0‐199)
Adenovirus	4217	710	1301	1487	719	27	(11‐61)
Influenza B	2095	25	697	1355	18	13	(0‐209)
Parainfluenza	1804	211	605	562	426	11	(2‐28)
Coronavirus	1591	253	524	562	252	10	(0‐52)
hMPV	1551	301	625	482	143	10	(0‐55)
All viruses	31 063	4521	11 091	10 821	4630	120	(38‐701)

^a^ wk: week number, and year, included in the study for the two incomplete seasons (2013/14 and 2016/17).

**Figure 2:**
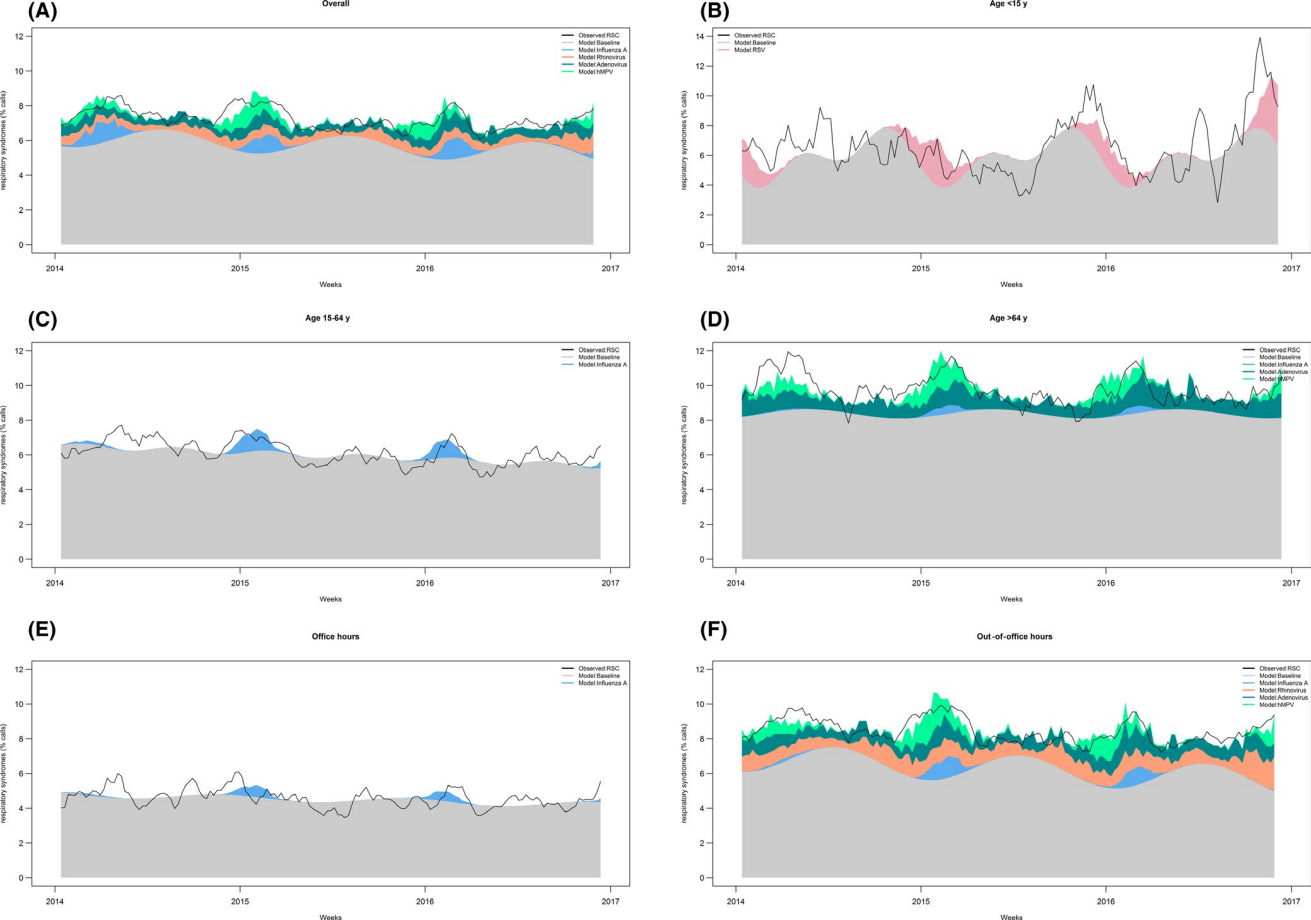
Results from the multivariate regression models: Stacked weekly respiratory syndrome calls (as proportion of all calls) attributed to different respiratory viruses. The black line represents the 5‐week moving average of the observed proportion of respiratory viruses and the coloured areas the proportions attributed by different viruses or to the unexplained baseline by the model

Associations between respiratory viruses and the proportion of RSC are reported in Table 4 and Figure 2. In children <15 years, only RSV was associated with RSC, explaining part of the RSC winter peaks, and being attributed 27 ambulance calls per year (3.8 per 100 000 inhabitants <15 years), which is around 11% of all RSC in this age group.

**Table 4:** Results from the multivariate models: associations between weekly numbers of positive laboratory tests for respiratory viruses and weekly proportion of ambulance dispatch calls due to respiratory syndromes (RSC)

	Respiratory viruses (×100)	Best fitting lag^f^	Coefficient (95% CI)	*P*‐value	Number of annual attributable RSC^*^	Proportion of all RSC^**^	RSC per 100 000 population per year
Overall^b^	Influenza A^a^ year 2013/14	+2	2.35 (0.90 to 3.83)	.0018	50 (19‐82)	1.12% (0.43‐1.83)	1.2 (0.4‐1.9)
	Influenza A^a^ year 2014/15		0.30 (0.08 to 0.52)	.0070	46 (13‐79)	0.86% (0.24‐1.49)	1.1 (0.3‐1.9)
	Influenza A^a^ year 2015/16		0.41 (0.16 to 0.66)	.0018	53 (20‐86)	1.02% (0.39‐1.66)	1.2 (0.5‐2.0)
	Influenza A^a^ year 2016/17		2.84 (−5.42 to 11.46)	.5095	71 (−135‐286)	1.32% (−2.53‐5.34)	1.7 (−3.2‐6.7)
	hMPV	−4	2.81 (1.00 to 4.62)	.0023	196 (70‐322)	3.82% (1.36‐6.29)	4.6 (1.6‐7.6)
	Adenovirus	−4	2.00 (0.51 to 3.50)	.0090	197 (27‐368)	3.84% (0.52‐7.17)	4.6 (0.6‐8.6)
	Rhinovirus	−1	1.04 (0.14 to 1.94)	.02365	335 (45‐627)	6.5% (0.89‐12.22)	7.9 (1.1‐14.7)
Age group <15 y^c^	RSV	−3	2.01 (0.06 to 4.07)	.0399	27 (0.8‐54)	10.59% (0.32‐21.45)	3.8 (0.1‐7.7)
Age group 15‐64 y^d^	Influenza A	−1	0.34 (0.16 to 0.53)	.0004	55 (25‐86)	2.56% (1.17‐3.98)	2.0 (0.9‐3.1)
Age group ≥65 y^e^	Influenza A	+2	0.14 (−0.23 to 0.51)	.4685	15 (−24‐54)	0.65% (−1.08‐2.40)	1.9 (3.1‐6.9)
	Adenovirus	+1	3.39 (0.65 to 6.16)	.0160	210 (40 −382)	9.33% (1.79‐16.97)	26.9 (5.1‐49.0)
	hMPV	−2	3.87 (0.58 to 7.19)	.0206	88 (13‐164)	3.92% (0.59‐7.29)	11.3 (1.7‐21.0)
Office hours^b^	Influenza A	−1	0.19 (−0.06 to 0.44)	.1356	19 (−6‐44)	0.05% (−0.02‐0.11)	0.4 (−0.1‐1.0)
Out‐of‐office hours^b^	Influenza A	+2	0.34 (0.10 to 0.58)	.0051	76 (23‐129)	1.47% (0.45‐2.51)	1.8 (0.5‐3.0)
	hMPV	−4	4.00 (1.96 to 6.04)	.0001	193 (94‐291)	3.76% (1.84‐5.68)	4.5 (2.2‐6.8)
	Rhinovirus	−1	1.88 (0.83 to 2.93)	.0005	419 (185‐654)	8.17% (3.60‐12.75)	9.8 (4.3‐15.4)
	Adenovirus	−4	2.90 (0.98 to 4.84)	.0031	380 (129‐634)	7.41% (2.52‐12.36)	8.9 (3.0‐14.9)

Note

Estimated coefficients have been multiplied by 100 to represent the increase in percentage points. When the effect was found to differ by epidemiologic year, epidemiologic year‐specific effects are shown. Coefficients indicate the increase in percentage points of calls that are respiratory syndromes per increase of 100 positive laboratory tests for respiratory viruses weekly.

^a^ The effect of Influenza A virus is presented stratified by epidemiological year.

^b^ Adjusted by sine and a cosine term with periodicity of 1 y and weekly linear trend.

^c^ Adjusted by sine and a cosine terms with periodicity of 1 y and half of a year.

^d^ Adjusted by sine and a cosine terms with periodicity of half of a year and weekly linear trend.

^e^ Adjusted by sine and a cosine term with periodicity of 1 y.

^f^ +lags mean that the RSC from the current week are best associated with viruses from *x *weeks in the past (ie trend in viruses precedes RSC); –lags mean that they are best associated with viruses from *x* weeks in the future (ie trend of RSC precedes the viruses).

* Calculated applying the model coefficient to the average weekly number of virus reports, and multiplied by the annual number of ambulance calls by epidemiologic year, age group, office or out‐of‐office hours, as appropriate; for the overall effects, this represents the average per epidemiologic year; for epidemiologic year‐specific effects, the numbers for incomplete epidemiologic years are extrapolations to represent complete epidemiologic years if the average weekly ILI incidence and ambulance calls were similar in non‐observed weeks than in observed weeks.

** Calculated dividing the number of RSC attributable to each virus (from the previous column) by the number of observed RSC by age group, epidemiologic year, office or out‐of‐office hours, as appropriate.

In the group 15‐64 years, only influenza A was associated with RSC, which explained 55 calls per year (2.0 calls per 100 000 inhabitants 15‐64 years), around 2.5% of all RSC in this age group. The effect did not differ by epidemiological year (interaction test *P* = .1625).

In people ≥65 years, RSC were associated with influenza A, hMPV and adenovirus. Visually, increases during winter peaks were attributable to influenza A and hMPV, while RSC attributable to adenovirus were reasonably constant throughout the year (Figure 2). Adenovirus had the biggest absolute impact, with 210 attributable calls per year, around 9% of all RSC in this group, while influenza A was attributed 15 per year, <1% of all RSC. The interaction between influenza A and epidemiologic year was statistically significant (*P* = .0079), but resulted in a negative coefficient for 2016/17 (stratified results therefore not presented).

In the overall sample, RSC were associated with the same viruses as observed in the people ≥65 with the addition of rhinovirus. The effect of influenza A was found to vary by epidemiologic year (*P* = .0178) and all coefficients were positive, although the association in 2016/17 was not statistically significant. However, the effect in absolute number of attributable RSC were similar by season, with around 50 RSC attributable to influenza A (1.2 per 100 000 inhabitants), only around 1% of all RSC. Rhinovirus was attributed the highest burden, with 6.5% of all RSC.

The results during out‐of‐office hours were mostly similar to the overall results with slightly higher proportions attributable to viruses, and the interaction by epidemiological year did not reach statistical significance (*P* = .1222). By contrast, the analysis of RSC during office hours failed to find any variability associated to respiratory viruses.

In most models, RSC were better associated with influenza A from 2 weeks previously, indicating that influenza A trends preceded RSC trends, except in the group 15‐64 years, were RSC preceded influenza A by 1 week (Table 4). RSC also preceded all other virus trends by 1‐4 weeks, except in the group ≥65 years, were adenovirus was found to precede RSC by 1 week.

## 4 DISCUSSION

Our results show that trends in RSC from highest urgency ambulance dispatches are associated with trends in the activity of common respiratory viruses. Depending on the subgroup 0%‐20% of RSC was attributable to a combination of respiratory viruses. The specific viruses contributing to RSC varied by age group, with estimates of 1%‐11% of RSC being attributable per individual virus. Their burden on these 4 call centres covering a 4 million population was 948 of highest urgency calls per year (22/100 000 inhabitants), although this varied by virus and age group.

In emergency departments, 25% of all acute respiratory diseases are attributable to respiratory pathogens,^14^ up to 80% in children.^15^ In our study, the majority of RSC were incorporated into the unexplained baseline. This is not an unexpected finding, since the categories of symptoms included in AMPDS triage codes are very broad, resulting in high background noise.^16^ Nevertheless, variability in RSC above this high baseline was associated with trends of common respiratory viruses, pointing at their potential usefulness to monitor the respiratory season overall (ie irrespective of the causative pathogen), as previously shown by its association with ILI.^12^ The different viruses potentially involved in RSC, their individual trends, and their seasonal variation in severity would make it challenging to design indicators and models that will allow us to prospectively use RSC data for situational awareness for specific viruses separately. Conversely, large or unexpected increases in a specific respiratory virus might be reflected to a certain extent in RSC.

Influenza A is a leading cause of acute lower respiratory tract infection, particularly in the elderly.^15^, ^16^ By contrast, in our study its contribution to RSC was low, especially among the elderly. In children, influenza was not associated to RSC, consistently with its low to marginal role in SARI in this age group.^13^, ^17^, ^18^, ^19^ The effect of influenza A on RSC (1%‐3%) is lower than what we found for ILI, which was attributed 4%‐34% of RSC.^12^ Influenza B did not show association with RSC in any group, in line with our understanding of its lower, less severe impact and lower clinical burden. Lower representativeness of the laboratory data in our current study may have underestimated the association for influenza, or oppositely, its effect estimated through ILI may be overestimated because ILI is caused also by other viruses.

The effect of influenza A on RSC was found to vary by season only for the overall sample. This is fundamental to assess whether these data can capture variations in severity of the circulating influenza strain, which is also likely to differ between age groups^4^ However, the season‐specific effects did not necessarily reflect the seasons known to have been more severe, although the interpretation is difficult, given that only two full seasons were included. Moreover, the specific effects in the incomplete seasons must be taken with caution. For year 2013/14, weeks 1‐26, 2014, overlapped with the entire influenza epidemic, possibly overestimating the effect of influenza, while for year 2016/17, weeks 27‐52, 2016, only captured the very beginning of the influenza epidemic, making it more difficult to establish associations.

The effects found for other viruses may be influenced by certain collinearity between them and the methodological choice of including influenza A a priori may affect their estimates. In the overall sample, rhinovirus showed the highest impact. Indeed, the role of rhinovirus in lower respiratory tract infections is increasingly established,^20^ and it is one of the most frequent viruses causing severe infections, second to RSV in children,^17^, ^18^, ^19^ and after influenza in adults.^15^, ^21^, ^22^, ^23^ Its presentation year‐round, with peaks in autumn and winter,^24^ also contributes to its high overall impact.

Adenovirus explained a significant proportion of RSC, especially among the elderly. Adenovirus is rarely detected in cases of severe respiratory infection,^15^, ^22^ although in a study in Finland, it was the second aetiology in mechanically ventilated patients with community‐acquired pneumonia.^21^ hMPV had a similar relative effect as adenovirus, although its impact on number of ambulance calls was smaller, since it was less frequent.

In children <15 years the peak in RSC developed earlier in the year, and our model associated this to RSV, consistent with its earlier presentation in the season.^13^, ^16^, ^25^ RSV is the leading cause of SARI in young children^13^, ^17^, ^18^, ^19^, ^23^ and has been highly associated to SARI syndromes in emergency departments^6^, ^14^ and ambulances.^10^, ^18^

The differences between office and out‐of‐office hours likely reflect that ambulance calls in these two time frames are distinct populations, probably with a different share of clinical pictures and severity. However, we cannot totally rule out a lack of statistical power during office hours, since the number of calls was smaller.

Ambulance dispatches are convenient for syndromic surveillance because they reflect events that are perceived as urgent (and thus potentially severe), they are recorded continuously and they have a virtually universal coverage.^8^, ^26^ Moreover, triage algorithms are increasingly standardized internationally.^5^, ^11^ However, the true usefulness and added value of ambulance dispatches for infectious disease surveillance needs to be studied and piloted prospectively. Some challenges for routinely using ambulance dispatch data prospectively include establishing data sharing routines and complying with data protection regulations.

There are limitations to our data. Because we did not include A2‐urgency level calls in our analysis, our results cannot be interpreted as the burden of respiratory viruses in ambulance services as a whole, but only in the highest urgency services. Since all associations are evaluated ecologically, spurious attribution of RSC trends to respiratory viruses cannot be ruled out. Sentinel laboratory surveillance has several limitations: it is passive and reported trends can include surveillance artefacts; it does not provide information on age, so overall number of virus detections was used; and while often biased to secondary care, it captures patients from both primary and secondary care, and the pathogens underlying their symptoms may differ from patients in ambulance dispatches. Our study covered only 6 RAVs, 25% of the population in the Netherlands, but we do not believe these are fundamentally different from non‐included RAVs. However, because the sentinel laboratory surveillance is widespread throughout the country, it could be possible that intensity or timeliness of circulation of the different viruses nationally is different from specific regional patterns in RAVs included in our study. Finally, as the Netherlands has a comprehensive primary care system where GPs that have a strong gate‐keeping role (including out‐of‐office services), our study results cannot be directly compared to health systems with higher use of emergency medical services.

## 5 CONCLUSION

Because of its ability to capture variations in respiratory virus circulation, ambulance dispatch data might be useful to signal events and to monitor the respiratory season as a whole, specifically reflecting severe infections and thus complementing existing surveillance systems. It will probably have less potential for drawing conclusions about the separate effect of specific individual viruses when not combined with information from other data sources, due to the low magnitude of some associations, the different viruses reflected in RSC and their proportional variation throughout the year. The true utility of ambulance dispatch data needs to be tested prospectively and faces potential challenges regarding timely data sharing and data protection.

## CONFLICT OF INTEREST

None declared.
